# The Impact of Childhood Attention-Deficit/Hyperactivity Disorder (ADHD) on Children’s Health-Related Quality of Life: A Systematic Review and Meta-Analysis

**DOI:** 10.1177/10870547231155438

**Published:** 2023-02-17

**Authors:** Sithara Wanni Arachchige Dona, Nalini Badloe, Emma Sciberras, Lisa Gold, David Coghill, Ha N. D. Le

**Affiliations:** 1 Deakin University, Burwood, VIC, Australia; 2 Royal Children’s Hospital, Parkville, VIC, Australia; 3 The University of Melbourne, Parkville, VIC, Australia

**Keywords:** HRQoL, ADHD, children and adolescents

## Abstract

**Objective:**

To investigate the association between children’s health-related quality of life (HRQoL) and childhood attention-deficit/hyperactivity disorder (ADHD).

**Method:**

Databases were systematically searched for peer-reviewed literature published between 2010 and 2022. Two reviewers independently screened and assessed the quality of included studies. Meta-analysis was conducted for studies that used the Pediatric Quality of Life Inventory (PedsQL).

**Results:**

Twenty-three studies were included, with most rated as “good” quality. Meta-analysis found “very large” effect in both parent- (Hedges’ g −1.67, 95% CI [−2.57, −0.78]) and child-reported (Hedges’ g −1.28, 95% CI [−2.01, −0.56]) HRQoL for children with ADHD compared to children without ADHD. No difference between parent- and child-reported HRQoL in children with and without ADHD was found. However, parent-reported HRQoL was lower than child-reported HRQoL among children with ADHD.

**Conclusion:**

ADHD was associated with substantially poorer children’s HRQoL. Among children with ADHD, parents rated their children’s HRQoL lower than the children themselves.

## Introduction

Childhood-onset attention-deficit/hyperactivity disorder (ADHD) is the most prevalent neurodevelopmental disorder globally (Asherson et al., 2016; Franke et al., 2018). ADHD is characterized by levels of inattention, impulsivity, and hyperactivity that are developmentally inappropriate and contribute to impairment in daily life (American Psychology Association, 2013; Costa Dias et al., 2013). The overall prevalence of ADHD in children was reported as 7.2% worldwide (Thomas et al., 2015). On average, children tend to be diagnosed with ADHD around the age of 7 in the US (Visser et al., 2014).

A robust body of evidence suggests that children with ADHD are at increased risk for other co-occurring conditions, including depression, anxiety, and substance use disorders (Asherson et al., 2016; Costa Dias et al., 2013). Additionally, ADHD is associated with lower educational or occupational achievement, reduced social functioning (Costa Dias et al., 2013; Franke et al., 2018), and increased risk of mortality driven by unnatural causes such as accidents (Dalsgaard et al., 2015), criminality (Dalsgaard et al., 2013), and suicidal behavior (Ljung et al., 2014). Evidence also suggests that children and adolescents with ADHD face significant stigmatization (Lebowitz, 2016) and increased victimization and bullying (Singh et al., 2010). On the other hand, there are ADHD-associated strengths related to children’s personality functions, such as high focus and energy, creativity, empathy, and agreeableness (Mahdi et al., 2017).

In recent years, the concept of quality of life (QoL) has become increasingly incorporated into health status evaluation as health-related quality of life (HRQoL) (D. Coghill et al., 2009; Danckaerts et al., 2010). While HRQoL and QoL have been used interchangeably (Karimi & Brazier, 2016), there are often confusions or misinterpretations on these terms (D. Coghill et al., 2009; Karimi & Brazier, 2016). In brief, QoL is a broad concept which measures someone’s perception of their life in terms of culture and value systems as well as about their standards, expectations, goals, and concerns (WHOQOL Group, 1995). On the other hand, the focus of HRQoL is on more the impacts of a disease or a health condition, and it does not measure some QoL aspects that are irrelevant to a health condition such as cultural and political attributes (Ferrans et al., 2005). As the most commonly accepted definition (Dey et al., 2012), HRQoL as a health status measure incorporates the holistic definition of health (World Health Organization, 2022) and provides insight into self-perceived wellbeing—that is, whether a patient “feels” healthy—and thus contributes to understanding the burden of a condition in terms of physical, psychological, and social functioning beyond simply symptomology (Celebre et al., 2021; D. Coghill et al., 2009; Dey et al., 2012).

HRQoL is, therefore, an important outcome measure and a meaningful opportunity to understand the impact of a condition on an individual’s life (Danckaerts et al., 2010; Dolan, 2000; Romero et al., 2013). Enhancing understanding of how conditions such as ADHD influence HRQoL can also provide directional insights for patient-centered service planning and supports to optimize outcomes for individuals (Danckaerts et al., 2010; Dolan, 2000; Romero et al., 2013; Sharpe et al., 2016).

When measuring overall functioning of children with ADHD, there are inconsistencies between HRQoL measurement tools as to how the domains are defined and what is included in each (D. Coghill et al., 2009; Danckaerts et al., 2010). Therefore, it can be difficult to corroborate and compare results from different HRQoL measurement tools. Both child self-reporting and parent-proxy reports have been used to measure HRQoL in children with ADHD, exhibiting a moderate agreement between these two raters (Bastiaansen et al., 2004). De Los Reyes et al. (2015) suggested that representation of a construct being measured in a child through parent- or teacher-reports should not be expected to be free from errors. Although parent-proxy reports may differ from children’s self-reported HRQoL in subjective experience and perception, they contribute to understanding HRQoL by providing complementary information (Bastiaansen et al., 2004; Danckaerts et al., 2010; Dey et al., 2012; Lee et al., 2016). Discrepancies between raters can be used to reveal key information regarding a child’s behavior in different contexts such as home or school, predicting poor outcomes on children, and identifying treatment outcome patterns (De Los Reyes, 2011). There is currently no agreed-upon “gold standard” for measuring children’s HRQoL, which makes it difficult to establish the validity of HRQoL tools (Danckaerts et al., 2010), particularly across a range of child age and cognitive abilities.

There are several previous systematic reviews exploring the impact of ADHD on children’s HRQoL (D. Coghill, 2010; Danckaerts et al., 2010; Galloway & Newman, 2017; Wehmeier et al., 2010) and two meta-analyses (Klassen, 2005; Lee et al., 2016). Specifically, these reviews found that ADHD significantly reduces children’s HRQoL, particularly from a parent’s perspective. Medication was associated with higher HRQoL in the short term (D. Coghill, 2010), while more significant social and emotional impairment was associated with lower HRQoL (Wehmeier et al., 2010). The impact of ADHD varies across domains of HRQoL, with a stronger association found for psychosocial than physical domains (Klassen, 2005; Lee et al., 2016). Some studies found disagreement between raters, with parent-proxy raters assigning lower HRQoL scores than children themselves (Danckaerts et al., 2010; Galloway & Newman, 2017), but Lee et al. (2016) found no difference between raters.

These reviews included literature published over 7 to 10 years ago. Given the growing research in this area over the past 10 years, an updated systematic review is needed to understand what is known more recently about the impact of ADHD on children and adolescents’ HRQoL. This knowledge will assist population health clinicians and policy-makers to effectively design interventions that can help improve children’s health and wellbeing among those living with ADHD. Therefore, we conducted a systematic review and a meta-analysis to synthesize the evidence on the relationship between childhood ADHD and children’s HRQoL, as well as the discrepancy of children’s HRQoL between parent- and child-reported HRQoL. We hypothesized that there is a negative association between childhood ADHD and children’s HRQoL.

## Methods

The systematic review adhered to and was reported based on the PRISMA 2020 guidelines (Page et al., 2021) and was registered in PROSPERO, ID number CRD42017071889 (Wanni Arachchige Dona et al., 2022).

### Identification of Studies

The following databases were systematically searched for literature published in English: MEDLINE, The Cochrane Library, EconLit, Embase, PsycINFO, CINAHL, DARE/NHSEED/HTA. As we aim to explore contemporary literature in the field of ADHD and children’s HRQoL, the search was limited to publications between 1st January 2010 and 15th July 2022. The search terms included various terms for children, ADHD, and HRQoL (see Appendix 1).

### Inclusion and Exclusion Criteria

Peer-reviewed studies were included if they: (1) focused on children and adolescents (between 0 and 18 years old), (2) included children with a formal diagnosis (that requires symptoms and impairment).Those with high symptoms for whom impairment was not formally assessed, we included studies that examined HRQoL in children (i) who met criteria for a diagnosis of ADHD which was diagnosed by any approach (e.g., previously diagnosed, diagnostic interview or above threshold on rating scales) or (ii) had high levels of ADHD symptoms rated using a validated rating scale, and (3) had a comparison group of children without ADHD. No restriction on HRQoL measures was applied. We considered all HRQoL measures, including ADHD-specific or generic measures, for example, Pediatric Quality of Life Inventory (PedsQL), EuroQoL-5D-Youth (EQ-5D-Y), and the Inventory of Quality of Life in Children and Adolescents (ILK). Studies were excluded if they did not meet the above criteria, or if they were: (1) not published in English, (2) published before 2010, (3) literature reviews, (4) qualitative studies, (5) grey literature, or (6) on non-human subjects.

### Study Selection and Data Extraction

All studies identified in the initial search underwent title and abstract screening by two reviewers (NB and SWAD) independently using Covidence (Veritas Health Innovation, 2020). Any disagreement between the reviewers resulted in the paper being retained for full-text screening. The remaining papers underwent full-text screening by the two reviewers (NB and SWAD) independently. Inter-rater reliability for the agreement between the two raters was tested using Cohen’s Kappa, resulting in a strong level of agreement (McHugh, 2012) at 0.81 and 0.87 Kappa value for title and abstract screening and full-text screening, respectively. Disagreements were discussed between the three reviewers (NB, SWAD, and HL) until a consensus was reached. One reviewer (NB) extracted data using MS Excel, which was then checked for accuracy by the other two reviewers (HL and SWAD), with an almost perfect level of agreement at 1.00 Kappa value for inter-rater reliability (McHugh, 2012). The data extracted included: the study population, the HRQoL instrument, whether the children’s HRQoL was self-reported or parent-reported, overall study results, and HRQoL scores (mean, SD), effect sizes, and *p* values where possible. The data were narratively synthesized by identifying common themes across studies following the guidelines by Braun and Clarke (2006).

### Quality Assessment

Quality assessment was conducted independently by two reviewers (NB and SWAD) on the included studies using the National Heart, Lung, and Blood Institute (NHLBI, 2014) quality assessment checklists for cohort, cross-sectional, and case-control studies. Any discrepancies were discussed and resolved between the three reviewers (NB, SWAD, and HL). As per previous literature using the same checklists (Gundmi et al., 2018; Sabeena et al., 2017), a “yes” option for each of the criteria was given a score of one, and the overall quality of studies was rated as “good,” “fair,” and “poor” when the overall score was ≥6, 4 to 5, and <4, respectively. Cohort, cross-sectional, and randomized controlled trials could receive a maximum score of 14, while the maximum score for case-control studies is 12. NHLBI allows to evaluate internal validity of studies by assessing the potential for bias where a “good” study represents the least risk of bias. The tool consists of 12 to 14 items assessing sources of bias such as patient selection, study power, and confounding (NHLBI, 2014). NHLBI has been widely used in previous literature reviews (Akpan et al., 2020; Gupta et al., 2017; Haighton et al., 2019; Ismaiel et al., 2021; Pires, 2022; Torres et al., 2020). Inter-rater reliability was conducted for the agreement between raters for each question of the assessment tools.

### Meta-Analysis (Quantitative Analysis)

Of studies that used the same HRQoL measures, most commonly used HRQoL measure (PedsQL) was included in meta-analysis to minimize errors in interpretation. Meta-analysis was conducted using MetaXL software version 5.3 on Excel (Epi Gear, 2022). An aggregated level of the meta-analysis was conducted to calculate the pooled effect sizes (standardized mean difference) using a more robust inverse variance heterogeneity (IVhet) model. The IVhet model retains a correct coverage probability and a lower observed variance than the random effect model estimates despite heterogeneity (Doi et al., 2015). Hedges’ g score was calculated to estimate the standardized mean difference (effect size). Multiple models (*n* = 4) were separately run using table parameters differently to compare: (1) parent-reported HRQoL between children with and without ADHD, (2) child-reported HRQoL between children with and without ADHD, (3) parent- and child-reported HRQoL for children with ADHD, and (4) parent- and child-reported HRQoL for the children without ADHD. For the above first and second analysis, a negative Hedges g indicated that children with ADHD have a lower HRQoL than children without ADHD, and a positive value indicates the opposite. For the above third and fourth analysis, a negative Hedges g indicated that parent-reported HRQoL was lower than child-reported. The effect size is interpreted as huge, very large, large, medium, small, and very small if (absolute value) Hedges’ g is at least 2.0, 1.2, 0.8, 0.5, 0.2, and 0.01, respectively (Sawilowsky, 2009). When 95% confidence intervals for the mean overlap between two groups, it is considered a non-significant difference.

Cochrane *Q* test and *I*^2^ test were conducted to assess homogeneity. *I*^2^ test was conducted to assess the percentage of true variation of included studies, and the *Q*-test to assess the variation in effect sizes in studies. A *p*-value less than .05 was regarded as statistically significant. *I*^2^ at least 25%, 50%, and 75% are considered low, moderate, and high heterogeneity, respectively (Higgins & Thompson, 2002). Publication bias was assessed using the Luis Furuya-Kanamori (LFK) index, which is related to the Doi plot (Furuya-Kanamori et al., 2018). An LFK index less than 1 shows no asymmetry, an index from 1 to 2 indicates a minor asymmetry, and an index of more than 2 suggests a major asymmetry (Furuya-Kanamori et al., 2018).

## Results

**Figure 1.**
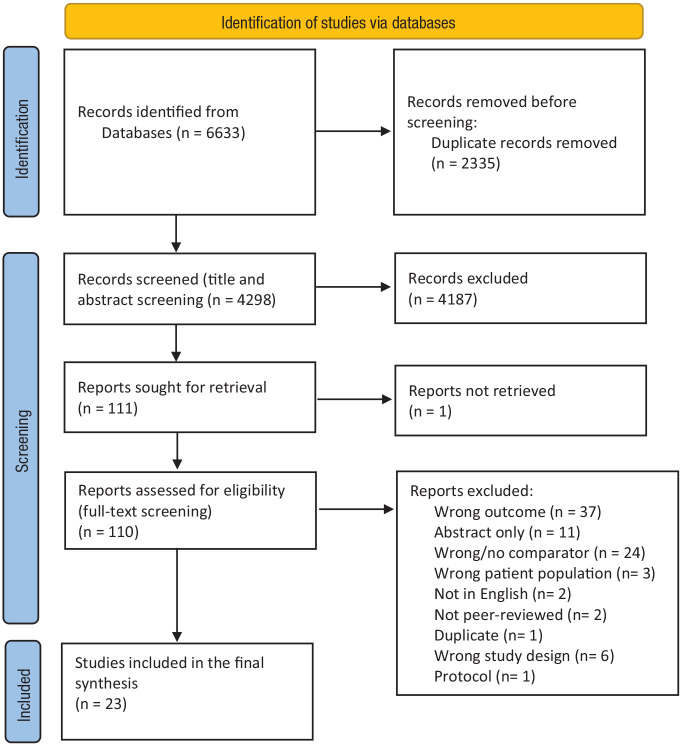
PRISMA diagram. *Source.* Adopted from: Page et al. (2021).

After duplicates were removed, 4,298 records were included in the title and abstract screening. Following this, 110 records underwent full-text screening, of which 23 were included for final synthesis (Figure 1). Table 1 presents the key characteristics of included studies, along with the quality assessment outcomes.

**Table 1.** Summary of Studies and Main Outcomes.

No.	Reference	Design	Country	HRQoL instrument	Sample (*n*); age	Outcomes in children with ADHD compared to children without ADHD	Quality of the study
*Parent-reported*							
1	Bai et al. (2017)	Cross-sectional	Netherlands	CHQ-PF28	ADHD *n* = 51; HC *n* = 4,539; 4–11-year-olds	Significant small effect on physical; physical score 59.93 (5.13) vs. 58.53 (4.28) for ADHD vs. control group*; large and significantly lower psychosocial scores; psychosocial score 46.57 (6.21) vs. 53.86 (5.87) for ADHD vs. control group	Good
2	Darweesh et al. (2021)	Cross-sectional	Egypt	PedsQL	ADHD *n* = 42; HC *n* = 42; 6–12-year-olds	Significant reduction in overall and individual domains; total score 125.76 (17.54) vs. 195.22 (3.81) for ADHD vs. control group; a correlation between subjective sleep problems/objective sleep parameters and PedsQL scores	Good
3	Green et al. (2016)	Cross-sectional	Australia	PedsQL	ADHD *n* = 164; HC *n* = 198; 6–10-year-olds	Significantly lower total and individual domain scores; total score 63.3 (13.7) vs. 81.7 (12.9) for ADHD vs. control group; ASD symptoms correlated with reduced HRQoL	Good
4	Grünwald and Schlarb (2017)	Cross-sectional	Germany	KINDL	ADHD *n* = 72; HC *n* = 79; 6–13-year-olds	Significantly lower HRQoL in children with all subtypes of ADHD; total score 69.39 (8.72) vs. 79 for ADHD-C vs. control group; reduced HRQoL in children with co-occurring sleep disorders than those without comorbidities	Good
5	Jamali et al. (2021)	Cross-sectional	Iran	POQL	ADHD/ODD *n* = 40; HC *n* = 80; 3–7-year-olds	Significantly higher dmft total scores; significantly poorer oral HRQoL; total score 0.096 (0.02) vs. 0.165 (0.01) for ADHD/ODD vs. control group	Good
6	Larsen et al. (2021)	Secondary analysis of RCT	Denmark	CHQ-PF28	ADHD *n* = 164 (pre and post); 3–7-year-olds	Lower total and psychosocial scores; score for psychosocial domain 38.07 (10.72) vs. 52.10 (7.90) for ADHD vs. control group	Good
7	Telman et al. (2017)	Cross-sectional	Netherlands	KIDSCREEN-27	ADHD-C *n* = 62; ADHD-I *n* = 64; HC *n* = 42; 6–21-year-olds (mean age 11.7)	Lower HRQoL scores across all domains, except for the school functioning; physical score 54.87 (11.12) vs. 57.77 (11.48) for ADHD-C vs. control group	Good
8	Maden and Gamlı (2022)	Cross-sectional	Turkey	The Turkish version of the ECOHIS (T-ECOHIS)	ADHD *n* = 76; HC *n* = 71; 6–13-year-olds	Significantly higher impact score, indicating poorer OHRQoL in children with ADHD than those without ADHD	Fair
*Child self-reported*							
9	Dewey and Volkovinskaia (2018)	Cross-sectional	Canada	KIDSCREEN-52; PRQ	ADHD *n* = 9; HC *n* = 16; DCD *n* = 9; ADHD + DCD *n* = 10; 11–18-year-olds	No significant difference in total HRQoL scores; 201.78 (25.62) vs. 212.69 (17.21) for ADHD vs. control group; significantly lower scores in mood and emotions and school domains in children with DCD + ADHD; high victimization correlated with reduced HRQoL	Good
10	Peasgood et al. (2016)	Cross-sectional	UK	CHU-9D; EQ-5D-Y/EQ-VAS	ADHD *n* = 476; HC *n* = 196 (SYC) + 4,234 (USoc); 6–18-year-olds	Significantly lower HRQoL across all domains, including happiness with life and family; 80.16 (20.61) vs. 86.93 (14.71) for ADHD vs. control group in EQ-VAS; significantly poorer outcomes in schoolwork, sleep problems, daily routine, feeling annoyed, and joining in activities	Good
11	Schwörer et al. (2020)	Cross-sectional	Germany	ILK	ADHD with drug treatment *n* = 58; ADHD without drug treatment *n* = 42; HC *n* = 100; 6–12-year-olds	Significantly poorer total HRQoL in children with ADHD without drug treatment than healthy controls, including school and family domains; lower social HRQoL in children with ADHD and executive function problems than healthy control; total score 4.10 (1.04) vs. 4.60 (0.68) for ADHD vs. control group	Good
12	Zambrano-Sánchez et al. (2012)	Prospective cohort	Mexico	AUQUEI	ADHD *n* = 120; HC *n* = 96; 7–12-year-olds	Significantly lower HRQoL scores; significantly lower scores in domains of family life and social relationships, and functions performance*; total score 45.2 vs. 54.3 for ADHD vs. control group	Good
*Combination of both parent- and child-reported*							
13	Becker et al. (2011)	Cross-sectional	Ireland	KINDL	ADHD *n* = 721 (pre and post); 6–17-year-olds	Significantly parent-reported lower scores on domains; total score 63.1 (13.3) vs. 66.6 (12.6) for parent vs. child rating	Good
14	Bussing et al. (2010)	Case-control	US	CIS; CHQ-PF28; YQOL	ADHD *n* = 169; HC *n* = 163; 6–17-year-olds	Significantly reduced parent-reported psychosocial scores; non-significant reduction in physical score; lower child-reported HRQoL; CIS score 51 vs. 32 for parents vs. child rating	Fair
15	D. Coghill and Hodgkins (2016)	Cross-sectional	Scotland	PedsQL; CHIP-CE	ADHD *n* = 213; HC *n* = 117; 6–16-year-olds	Reduced parent and child reported HRQoL; lower HRQoL with increased age, lower SES, and ADHD symptom severity; total score 62.1 (16.5) vs. 72.1 (15.5) for parents vs. child rating	Good
16	Göker et al. (2011)	Cross-sectional	Turkey	CQLS; Rosenburg Self Esteem Scale	ADHD *n* = 50; HC *n* = 30; 7–15-year-olds	Significantly lower parent-reported total and individual domain scores; no significant difference in child-reported HRQoL between children with and without ADHD, except school domain; significantly lower HRQoL in children with ADHD and low self-esteem than healthy control with low self-esteem; total score 56.7 (15.6) vs. 75.1 (15.8) for parents vs. child rating	Good
17	Jafari et al. (2011)	Cross-sectional	Iran	PedsQL	ADHD *n* = 72; HC *n* = 140; 8–18-year-olds	Significantly lower parent and child-reported total and individual scores (except parent-reported social domain); higher child scores than parents in children with and without ADHD; total score 57.06 (14.67) vs. 62.39 (17.20) for parents vs. child rating	Good
18	Kandemir et al. (2014)	Cross-sectional	Turkey	PedsQL	ADHD *n* = 76; HC *n* = 59; 7–16-year-olds	Significantly lower parent and child-reported total and psychosocial scores; total score 69.06 (14.32) vs. 72.13 (15.02) for parents vs. child rating	Good
19	Limbers et al. (2011)	Cross-sectional	US	PedsQL; PedsQL Family	ADHD *n* = 196; HC *n* = 876; 5–18-year-olds	Significantly lower parent and child-reported total and individual domain scores, particularly child-reported school and parent-reported psychosocial score; total score 69.88 (15.91) vs. 73.91 (17.19) for parents vs. child rating	Good
20	Marques et al. (2013)	Cross-sectional	Brazil	PedsQL	ADHD *n* = 45; HC *n* = 43; 8–12-year-olds	Significantly lower parent-reported total and individual domain scores; child-reported lower scores in all domains, but statistically significant lower scores in total, psychosocial, school, and social functioning; total score 67.10 (2.21) vs. 69.15 (2.37) for parents vs. child rating	Good
21	Thaulow and Jozefiak (2012)	Cross-sectional	Norway	ILK; C-GAS	ADHD *n* = 62; Anxiety/depression *n* = 49; HC *n* = 65; 8–15.5-year-olds	Significantly lower parent and child-reported HRQoL; significantly higher HRQoL in children with ADHD than children with anxiety or depression disorders; higher child-reported HRQoL than parent-reported scores in children with ADHD; total score 73.6 (14.9) vs. 62.5 (13.8) for parent vs. child rating	Fair
22	Velő et al. (2021)	Cross-sectional	Hungary	ILK	ADHD *n* = 79; HC *n* = 54; 6–18-year-olds	A significant negative weak correlation between peer relationships and child-reported HRQoL only in children with ADHD; a significant negative moderate correlation between peer relationships and parent-reported HRQoL in both children with ADHD and healthy control; total score 28.12 (5.40) vs. 29.17 (3.85) for parents vs. child rating	Good
23	Yürümez and Kılıç (2016)	Cross-sectional	Turkey	PedsQL	ADHD *n* = 46; HC *n* = 31; 7–13-year-olds	Significantly higher frequency of sleep problems; lower parent and child-reported HRQoL scores, even after correcting for having/not having sleep problems; total score 65.2 (15.1) vs. 68.8 (16) for parents vs. child rating	Fair

*Note.* ADHD = Attention deficit hyperactivity disorder; HC = Healthy Control (Children without ADHD); HRQoL = Health-related Quality of life; ASD = autism spectrum disorder; AUQUEI = Auto Questionnaire Qualité de Vie-Enfant-Imagé; C-GAS = Children’s Global Assessment Scale; CHIP-CE = Child Health & Illness Profile-Child Edition; CHQ-PF28 = Child Health Questionnaire Parent Form28; CHU-9D = Child Health Utility 9D; CIS = Columbia Impairment Scale; CQLS = Children’s Quality of Life Scale; DCD = Developmental Coordination Disorder; dmft = decayed missing filled teeth; EQ-5D-Y = EuroQoL-5D-Youth; ILK = the Inventory of Quality of Life in Children and Adolescents; KINDL = *a* generic instrument to measure the quality of life in children; LIS = Impairment scale developed for Lifetime Impairment Survey; ODD = Oppositional Defiant Disorder; PedsQL = the Pediatric Quality of Life Inventory; POQL = Pediatric Oral Health-related Quality of Life; PRQ = Peer Relations Questionnaire; RCT = Randomized Control Trial; SES = Socioeconomic Status; SYC = South Yorkshire Cohort; USoc = Understanding Society: The UK’s Household Longitudinal Survey; YQOL = Youth Quality of Life questionnaire.

## Study Characteristics

There were a broad range of different HRQoL instruments used in included studies such as the Child Health Questionnaire (CHQ) (*n* = 3), the Inventory of Quality of Life in Children and Adolescents (ILK) (*n* = 3), KINDL (*n* = 2), and the KIDSCREEN instrument (*n* = 2). The most commonly used HRQoL instrument was PedsQL (*n* = 8) (Table 1).

Among 23 included studies, most were cross-sectional (*n* = 20), with one cohort study, one case-control study, and one secondary analysis of a randomized controlled trial. Ten studies were from Europe, seven from the Middle East-North Africa region, five from the Americas, and one from Australia (Table 1). Half of the studies (*n* = 11) used both parent- and child-reported HRQoL measures, four studies used only child-reported, and eight relied solely on parent-reported HRQoL measures. Studies included children who were clinically diagnosed for ADHD (*n* = 18), or reported symptoms (presence of at least three main diagnostic criteria of the Diagnostic and Statistical Manual of Mental Disorders [DSM]) by their parents (*n* = 1) or categorized using various tools (e.g., DSM, parent questionnaires, and school report) by researchers (*n* = 4). Of 18 studies that included children with clinical diagnosis, the DSM-IV or V was the most common approach (*n* = 13 studies) to clinical diagnosis of ADHD. The quality of the majority of the studies (*n* = 19, 83%) was rated as “good.” Only four studies were rated as “fair,” and none were of “poor” quality (Appendix 2.1). Inter-rater reliability for the agreement between two raters was in a range of strong (0.80) to almost perfect (1.00) (Appendix 2.2).

## Impact of ADHD on Children’s HRQoL

### Narrative Synthesis

#### Self-Reported HRQoL of Children With ADHD Compared to Children Without ADHD

Child-reported overall HRQoL was found to be lower in children with ADHD than children without ADHD in all included studies, except one (Dewey & Volkovinskaia, 2018).

In regards to the association between ADHD and HRQoL domains, studies using the same child-reported HRQoL measure (i.e., PedsQL) (*n* = 6) found significantly lower HRQoL scores in the school and psychosocial domains (including social, emotional, and school domains) among children with ADHD compared to children without ADHD (D. Coghill & Hodgkins, 2016; Jafari et al., 2011; Kandemir et al., 2014; Limbers et al., 2011; Marques et al., 2013; Yürümez & Kılıç, 2016). Studies using other instruments also found consistently lower domain HRQoL scores in related domains (e.g., family life and social relationship) among children with ADHD than children without ADHD (Bussing et al., 2010; D. Coghill & Hodgkins, 2016; Dewey & Volkovinskaia, 2018; Göker et al., 2011; Peasgood et al., 2016; Schwörer et al., 2020; Thaulow & Jozefiak, 2012; Velő et al., 2021; Zambrano-Sánchez et al., 2012).

There are mixed findings in relation to the association between ADHD and physical domains of HRQoL. Five studies indicated that children with ADHD did not report any significant difference in the physical domain compared to children without ADHD (Bussing et al., 2010; Dewey & Volkovinskaia, 2018; Göker et al., 2011; Kandemir et al., 2014; Marques et al., 2013) while eight studies found significantly lower scores in physical domains in children with ADHD than children without ADHD (Becker et al., 2011; D. Coghill & Hodgkins, 2016; Jafari et al., 2011; Limbers et al., 2011; Peasgood et al., 2016; Thaulow & Jozefiak, 2012; Velő et al., 2021; Yürümez & Kılıç, 2016).

Among children with ADHD, children with ADHD treatment had higher HRQoL than those without treatment but even with medication treatment, children with ADHD still had lower HRQoL in the family domain than those without ADHD (Schwörer et al., 2020).

#### Parent-Reported HRQoL of Children With ADHD Compared to Children Without ADHD

A total of 19 studies used parent-proxy HRQoL measures. Similar to child-reported HRQoL, parents of children with ADHD consistently rated their child’s HRQoL lower than children without ADHD in all included studies (see Table 1). The association between ADHD and lower parent-reported HRQoL domain scores were also reported in the emotional, school, and psychosocial HRQoL domains (measured by the PedsQL) (Darweesh et al., 2021; Kandemir et al., 2014; Larsen et al., 2021; Limbers et al., 2011; Marques et al., 2013; Telman et al., 2017) and in achievement and risk avoidance (assessed using the Child Health & Illness Profile-Child Edition [CHIP-CE]) (D. Coghill & Hodgkins, 2016) in children with ADHD compared to children without ADHD.

Again, parent-proxy reports of HRQoL yielded inconsistent results for the physical domains of HRQoL among children with ADHD. The majority of studies (*n* = 12) found a significant reduction in physical HRQoL among children with ADHD than children without ADHD (Becker et al., 2011; D. Coghill & Hodgkins, 2016; Darweesh et al., 2021; Göker et al., 2011; Green et al., 2016; Grünwald & Schlarb, 2017; Jafari et al., 2011; Limbers et al., 2011; Telman et al., 2017; Thaulow & Jozefiak, 2012; Yürümez & Kılıç, 2016). However, Bai et al. (2017) found that parents reported a higher physical HRQoL on the Child Health Questionnaire (CHQ) for children with ADHD than for children without ADHD, and the authors gave the highly active nature of the condition as a possible reason. Other studies (*n* = 4) found no significant difference in parent-reported physical HRQoL in children with ADHD compared to children without ADHD (Bussing et al., 2010; Kandemir et al., 2014; Larsen et al., 2021; Marques et al., 2013).

Jamali et al. (2021), who examined the oral HRQoL among children with ADHD compared to children without ADHD, found significantly more decayed, missing, or filled teeth among children with ADHD than children without ADHD, and significantly lower scores across all domains of oral HRQoL (Jamali et al., 2021). Similarly, Maden and Gamlı (2022) reported poorer oral HRQoL in children with ADHD than those without ADHD.

#### Agreement Between Child- and Parent-Reported HRQoL

There were mixed findings on the agreement between parent- and child-reported HRQoL across studies. Marques et al. (2013) found a greater agreement between parent- and child-reported HRQoL among those with ADHD compared to children without ADHD across all domains except school functioning. D. Coghill and Hodgkins (2016) reported a significant correlation between parent and child ratings for PedsQL and CHIP-CE measures with low to moderate strength among all children. On the other hand, other studies found that children with ADHD rated their HRQoL higher than parent ratings (Göker et al., 2011; Jafari et al., 2011; Thaulow & Jozefiak, 2012).

### Quantitative Analysis (Meta-Analysis) Findings

To minimize the difficulties resulting from pooling data and interpreting results of various HRQoL measures, studies that used the same HRQoL measure (i.e., PedsQL) reporting results in a consistent way were included in the meta-analysis (*n* = 8). Eight studies used a parent-reported measure and six used both parent and child-reported HRQoL measures using PedsQL. When the HRQoL of the children with ADHD was compared with more than one group, only comparison data from the group of children without ADHD were used.

#### Overall HRQoL

The pooled effect (the standardized mean difference) of ADHD on parent-reported children’s overall HRQoL for children with ADHD compared to children without ADHD was “very large” (Hedges’ g −1.67, 95% CI [−2.57, −0.78]). Similarly, the effect of ADHD on child-reported children’s HRQoL was also “very large” (Hedges’ g −1.28, 95% CI [−2.01, −0.56]) for children with ADHD compared to children without ADHD (see Appendix 3 for forest plots). The negative Hedges’ g values indicated that children with ADHD had a worse overall HRQoL than children without ADHD from both parent’s and children’s perspectives. There was no significant difference between parent-reported and child-reported HRQoL (i.e., there is an overlap between the 95% confidence interval of parent-reported [−2.57, −0.78] and child-reported children’s overall HRQoL [−2.01, −0.56]) when analyzing children with and without ADHD together.

However, for the subgroup of children with ADHD, there was a statistically significant difference between parent and child ratings, with parent ratings of their children’s HRQoL lower than the children’s ratings of their own HRQoL (Hedges g −0.49, 95% CI [−0.68, −0.31]) (Appendix 4). For the subgroup of children without ADHD, there was no significant difference between parent and child ratings (Hedges’ g 0.005, 95% CI [−1.08, 1.09]) (Appendix 5).

#### Individual Domains of Children’s HRQoL

We found a “medium” (Hedges’ g −0.79, 95% CI [−1.14, −0.45]) to “large” (Hedges’ g −0.88, 95% CI [−1.48, −0.29]) impact of ADHD on the physical domain in child- and parent-reported, respectively. A “very large” impact on psychosocial domain was found in both parent- (Hedges’ g −1.96, 95% CI [−3.24, −0.68]) and child-reported (Hedges’ g −1.42, 95% [CI −2.53, −0.32]) scorings (see Appendices 3.2 and 3.3). The impact of ADHD on the school domain was “very large” (Hedges’ g −1.29 and −1.79 for child- and parent-reported).

There was no significant difference between parent- and child-reported HRQoL for all individual domains across all children (Appendix 3). However, in the subgroup of children with ADHD, the difference between parent and child ratings of HRQoL was statistically significant for the physical (Hedges’ g −0.23, 95% CI [−0.37, −0.08]), psychosocial (Hedges’ g −0.50, 95% CI [−0.89, −0.11]), and emotional (Hedges’ g −0.52, 95% CI [−0.79, −0.25]) domains, but not for the other sub-domains of the psychosocial domain (i.e., school and social domains).

#### Heterogeneity and Publication Bias

Heterogeneity in effect sizes of difference between children with and without ADHD was significant for overall HRQoL from both parent (Q 245.47, *p* < .05) and child perspectives (Q 96.11, *p* < .05). The *I*^2^ test indicated a high heterogeneity at 97 and 95 for both parent and child ratings (*I*^2^ > 75%) (Appendix 6). The Luis Furuya-Kanamori (LFK) index indicated a major asymmetry for parent-reported overall, and psychosocial domains and a minor asymmetry for physical domain due to publication bias. Similarly, the LFK index for child-reported emotional, social, and school domains showed a major asymmetry and a minor asymmetry for overall and physical domains due to publication bias (Appendix 6).

## Discussion

HRQoL is increasingly valued as a key element to understanding the impact of health problems on children, particularly with mental health issues (D. Coghill et al., 2009; Danckaerts et al., 2010) and is increasingly considered to be crucial to measure as a treatment outcome (Adamo et al., 2015). Given that previous reviews included literature published more than 7 to 10 years ago and the growing research in this area, this systematic review has synthesized contemporary literature (i.e., published since 2010) relating to the impact of ADHD on children’s HRQoL to better understand the impact of the condition. Consistent with previous reviews (D. Coghill, 2010; Danckaerts et al., 2010; Galloway & Newman, 2017; Klassen, 2005; Lee et al., 2016; Wehmeier et al., 2010), we found that both the children themselves and their parents reported poorer HRQoL in children with ADHD compared to children without ADHD. There was no difference between child and parent ratings of children’s HRQoL when HRQoL data from all children (with and without ADHD) were considered together. However, for the subgroup of children with ADHD, parents rated their child’s HRQoL lower than the children themselves.

The large number of studies that included child self-reported HRQoL measures suggests a positive growth in the use of individual self-perception instruments as a measure of child’s HRQoL. This is consistent with previous systematic reviews in this area which have found that child self-reporting is not interchangeable to parent-proxy ratings of child’s HRQoL (Danckaerts et al., 2010; Galloway & Newman, 2017). The differences in the agreement between parent- and child ratings of HRQoL in the included studies indicate the importance of including both proxy- and self-reported measures of HRQoL in research (D. Coghill et al., 2009; Dey et al., 2012; Jonsson et al., 2017). This is the approach used in half of included studies (11/23) in our review, which is much higher than the proportion of studies including both proxy-and self-rating HRQoL measures in previous reviews, for example, 4/16 in D. Coghill (2010) and 4/20 in Danckaerts et al. (2010).

There are possible explanations for the differences in parent and child rating. For example, Klassen et al. (2006) mentioned that ADHD symptoms and the associated impulsive cognitive style could influence how a child may complete a HRQoL questionnaire, highlighting the conceptual challenges of measuring HRQoL among children with ADHD. Waters et al. (2003) stated that the difference could be due to various health, social, and educational factors, such as parents with health issues or low sociodemographic status. De Los Reyes and Kazdin (2005) developed a theoretical framework on raters’ discrepancies in clinical research and provided attributions and perspectives as factors for child-parent discrepancies. For example, they proposed that as per parents, children’s disposition (i.e., being aggressive) contributes to their problems (e.g., aggressive behavior, hyperactivity, depression), while children often see the context or environment (i.e., protecting themselves from peers) as attributions to their problems, resulting in differences in ratings.

HRQoL is by definition self-evaluative, making self-reported instruments crucial in researching HRQoL outcomes (Varni & Burwinkle, 2006). There are, however, benefits and insights to be gained from proxy reports (Varni et al., 2007). In particular, when a child is too young, cognitively impaired, or severely ill, parent proxy reports are a reliable and valid option (Varni et al., 2007). Moreover, parents’ perception is crucial in health service utilization as they make decisions on child’s health in seeking care and treatment (Cremeens et al., 2006). Seeking treatments should be promoted as there are short-term and long-term positive effects of treatments on HRQoL in children with ADHD (D. Coghill, 2010).

Despite some differences between self-reported and parent proxy-report, the studies included in this review consistently demonstrated that regardless of self- or parent-proxy reporting, children and adolescents with ADHD experience a poorer overall HRQoL, particularly in the psychosocial domains, compared to children without ADHD. These findings align with previous reviews (Danckaerts et al., 2010; Jonsson et al., 2017; Lee et al., 2016; Orm & Fjermestad, 2021; Wehmeier et al., 2010). Larsen et al. (2022) found that children with ADHD are prone to frequent and severe functional somatic symptoms (e.g., stomach pain, tiredness, and headache), resulting in poor HRQoL with more emotional and behavioral difficulties than those without ADHD. Riley et al. (2006) reported that children’s HRQoL can be deteriorated because of ADHD symptoms, co-occurring health conditions (e.g., autism spectrum disorder), issues with peers, conduct and co-ordination as well as family issues such as parental sickness and behaviors (e.g., depressions/stress, maternal smoking) and family composition (e.g., divorced, living separately). Understanding these contributing factors to poor HRQoL is important to evaluate and improve treatment options for ADHD in children.

Our meta-analysis confirmed the large impact of ADHD on children and adolescents from both parents’ and children’s perspectives. Findings on the agreement in parent-reported and child-reported overall and individual domains of children’s HRQoL are consistent with the previous meta-analysis on HRQoL in children and adolescents with ADHD (Lee et al., 2016). Consistent with Lee et al. (2016)’s study, we found a “very large” impact of ADHD on psychosocial domain, with a “very large” impact on the school, emotional and social sub-domains. This evidence is consistent with literature exploring the impact of ADHD on academic performance (Arnold et al., 2020; Taanila et al., 2014) and highlights a need to provide adequate educational and behavioral support for children with ADHD at school. Population health interventions for ADHD also need to incorporate educational support services in addition to the pharmacological and behavioral interventions.

Consistent with previous reviews, we found that ADHD impacted more on the psychosocial domains than physical domains of HRQoL. However, slightly different to Lee et al. (2016)’s meta-analysis who found a moderate impact on physical domain, our meta-analysis found a “medium” and a “large” impact of ADHD on the physical domain respectively in child- and parent-reported HRQoL. It should be noted that our review and meta-analysis included more recent studies (from 2010 onward) while Lee et al. (2016)’s included studies prior to 2014, with only three studies common in both meta-analyses. Consistent with Lee et al. (2016), substantial heterogeneity was also found across studies which could be due to differences in study design and study participants. For example, four out of eight studies included in the meta-analysis included children with co-occurring conditions (e.g., ODD) which could have impacted these children’s HRQoL above and beyond ADHD itself (D. Coghill & Hodgkins, 2016; Limbers et al., 2011; Marques et al., 2013; Yürümez & Kılıç, 2016).

We extended the previous meta-analysis by exploring the agreement of self and proxy HRQoL reporting in the subgroup of children with ADHD. We found a significant difference between parent and child reporting on overall HRQoL as well as in the physical and emotional QoL domains, but no differences in the school and social domains. Consistent with Lee et al. (2016), this finding shows the importance of assessing children’s and adolescent’s HRQoL with ADHD using both parent proxy- and child self-reporting.

Strengths of this study include the broad search of the contemporary literature on the impact of ADHD on children’s HRQoL. Some limitations are noted. First, studies published in languages other than English, prior to 2010 and grey literature were not considered, and the review may have missed some literature due to this exclusion. Second, given that the studies included in the meta-analysis did not provide segregated PedsQL scoring for individual age groups or gender, subgroup analysis by age or gender could not be conducted. Given that age was associated with all domains of HRQoL as shown in Lee et al. (2016), further research should explore the age or gender effect on HRQoL among children with ADHD.

Findings about the large impact of ADHD on children’s HRQoL in our review and meta-analysis highlight a need for effective treatment (e.g., pharmacological [D. R. Coghill et al., 2017]) and behavioral treatment) and adequate support for these children. Children and adolescents with ADHD also experience poorer functional outcomes, which has been well-documented in previous literature as well as being an established diagnostic criterion for ADHD diagnosis (Cândido et al., 2021; Garner et al., 2013; Weiss, 2015). Furthermore, an ADHD diagnosis can increase the likelihood of experiencing victimization and bullying for a child, possibly due to the social stigma associated with ADHD (Lebowitz, 2016; Singh et al., 2010). It is, therefore, important to acknowledge the broader impact of ADHD on children and adolescent’s daily functionality so that sufficient support could be provided for children with ADHD. Given the school domain is severely impacted by ADHD, as evidenced in our meta-analysis, educational support for children with ADHD is essential.

Improving children’s HRQoL is increasingly identified as a key goal of ADHD treatment alongside clinical treatment outcomes and improvement in functional outcomes (Danckaerts et al., 2010), thus, routine inclusion of a generic HRQoL measure in health interventions for children with ADHD is encouraged. Future research should also incorporate both parent and child perspectives in HRQoL measures as both appear to be important in understanding the broad impact of ADHD on the child’s everyday life functioning/wellbeing.

## Conclusion

This systematic review and meta-analysis found that children with ADHD had significantly poorer HRQoL than children without ADHD from both parents’ and children’s perspectives. The impact of ADHD on children’s HRQoL appeared to be “very large” for both parent- and child-reported HRQoL. There were no significant differences in parent-reported and child-reported HRQoL across all children. However, for the children with ADHD, there was a statistically significant difference between parent and child reporting on overall HRQoL, physical and psychosocial domains.

These findings highlight the need for effective ADHD treatment and for increased efforts to make ADHD treatment accessible for families. Strategies to treat or support children with ADHD should consider the child’s wellbeing rather than only the condition itself. Future studies exploring HRQoL in children with ADHD may consider including both parent- and child-reported HRQoL measures.

## Supplemental Material

sj-docx-1-jad-10.1177_10870547231155438 – Supplemental material for The Impact of Childhood Attention-Deficit/Hyperactivity Disorder (ADHD) on Children’s Health-Related Quality of Life: A Systematic Review and Meta-Analysis Click here for additional data file. Supplemental material, sj-docx-1-jad-10.1177_10870547231155438 for The Impact of Childhood Attention-Deficit/Hyperactivity Disorder (ADHD) on Children’s Health-Related Quality of Life: A Systematic Review and Meta-Analysis by Sithara Wanni Arachchige Dona, Nalini Badloe, Emma Sciberras, Lisa Gold, David Coghill and Ha N. D. Le in Journal of Attention Disorders
