# Supplementary material for: The Impact of Childhood Attention-Deficit/Hyperactivity Disorder (ADHD) on Children’s Health-Related Quality of Life: A Systematic Review and Meta-Analysis
Source: J Atten Disord. 2023 Feb 17;27(6):598–611. doi: 10.1177/10870547231155438 (PMC10068409; doi:10.1177/10870547231155438)
Supplement: sj-docx-1-jad-10.1177_10870547231155438 – Supplemental material for The Impact of Childhood Attention-Deficit/Hyperactivity Disorder (ADHD) on Children’s Health-Related Quality of Life: A Systematic Review and Meta-Analysis [file sj-docx-1-jad-10.1177_10870547231155438.docx]

***Appendices***

**Appendix 1: Search strategy**

| S1 | child* OR adolescen* OR "young people" OR teen* OR youth* OR pediatri* OR paediatri* |
| --- | --- |
| S2 | ("ADHD" OR "ADD" OR "attention deficit hyperactivity disorder" OR "attention deficit disorder" OR "attention deficit disorder with hyperactivity") OR attenti* adj3 (defic* or dysfunc* or disorder* OR hyperactiv* OR hyperkinesis*) |
| S3 | wellbeing OR 'well being' OR 'QOL' OR 'quality of life' OR 'HRQOL' OR 'personal satisfaction' OR 'health status' OR 'functional status' OR 'self-esteem' OR 'self concept' OR 'self competence' |
| S4 | S1 AND S2 AND S3 |

**Appendix 2.1: Quality Assessment**

| **Study** | **Criteria** | | | | | | | | | | | | | | |
| --- | --- | --- | --- | --- | --- | --- | --- | --- | --- | --- | --- | --- | --- | --- | --- |
|  | **1** | **2** | **3** | **4** | **5** | **6** | **7** | **8** | **9** | **10** | **11** | **12** | **13** | **14** |  |
| **Cross-sectional** | | | | | | | | | | | | | | | |
| Bai et al. (2017) | Y | Y | Y | Y | N | NA | NA | N | Y | NA | Y | NA | NA | Y | good |
| Becker et al. (2011) | Y | Y | Y | CD | N | NA | NA | Y | Y | NA | Y | NA | CD | N | good |
| Coghill & Hodgkins (2016) | Y | Y | Y | Y | Y | Y | NA | Y | Y | NA | Y | NA | NA | Y | good |
| Dewey & Volkovinskaia (2018) | Y | CD | Y | Y | N | Y | NA | N | Y | NA | Y | NA | NA | N | good |
| Göker et al. (2011) | Y | Y | CD | Y | N | NA | NA | N | Y | NA | Y | NA | NA | Y | good |
| Green et al. (2016) | Y | Y | CD | CD | N | NA | NA | Y | Y | NA | Y | NA | NA | Y | good |
| Grünwald & Schlarb (2017) | Y | Y | CD | Y | N | Y | NA | Y | Y | NA | Y | NA | NA | N | good |
| Jafari et al. (2011) | Y | Y | CD | Y | N | Y | NA | Y | Y | NA | Y | NA | NA | N | good |
| Jamali et al. (2021) | Y | Y | CD | Y | N | Y | NA | N | Y | NA | Y | NA | NA | Y | good |
| Kandemir et al. (2014) | Y | Y | CD | Y | N | Y | NA | N | Y | NA | Y | NA | NA | N | good |
| Limbers et al (2011) | Y | Y | CD | CD | N | Y | NA | Y | Y | NA | Y | NA | NA | N | good |
| Marques et al. (2013) | Y | Y | CD | Y | N | NA | NA | Y | Y | NA | Y | NA | NA | N | good |
| Peasgood et al. (2016) | Y | Y | Y | Y | N | Y | NA | N | Y | NA | Y | NA | NA | Y | good |
| Schwörer et al. (2020) | Y | Y | Y | Y | N | Y | NA | N | Y | NA | Y | NA | NA | Y | good |
| Telman et al. (2017) | Y | Y | Y | Y | N | Y | NA | Y | Y | NA | Y | NA | NA | Y | good |
| Thaulow & Jozefiak (2012) | Y | Y | CD | CD | N | NA | NA | Y | Y | NA | Y | NA | NA | N | Fair |
| Velo et al. (2021) | Y | Y | Y | Y | N | NA | NA | Y | Y | NA | Y | NA | NA | N | good |
| Yürümez & Kılıç (2013) | Y | Y | CD | Y | N | NA | NA | N | Y | NA | Y | NA | NA | N | Fair |
| Maden & Gamli (2022) | Y | Y | NR | Y | N | Y | NA | N | N | NA | Y | NA | NA | N | Fair |
| **Cohort studies** | | | | | | | | | | | | | | | |
| Zambrano-Sánchez et al. (2012) | Y | Y | CD | Y | N | NA | NA | N | Y | NA | Y | NA | NA | Y | Good |
| **RCT** | | | | | | | | | | | | | | | |
| Larsen et al. (2021) | Y | CD | N | N | N | Y | Y | Y | CD | CD | Y | N | Y | Y | Good |

Note: the criteria were obtained from NHLBI tool for cohort and cross-sectional studies, and for controlled intervention studies; Y= Yes, N= No, CD= Cannot determine, NA= Not applicable, NR= Not reported

|  | **QA Criteria** | | | | | | | | | | | |  |
| --- | --- | --- | --- | --- | --- | --- | --- | --- | --- | --- | --- | --- | --- |
| **Study** | **1** | **2** | **3** | **4** | **5** | **6** | **7** | **8** | **9** | **10** | **11** | **12** |  |
| **Case-control** | | | | | | | | | | | | | |
| Bussing et al. (2010) | Y | N | N | Y | NR | Y | NR | CD | CD | Y | N | Y | Fair |
| Darweesh et al. (2021) | Y | Y | Y | Y | Y | Y | NA | CD | Y | Y | N | Y | good |

Note: the criteria were obtained from NHLBI tool for case-control studies; Y= Yes, N= No, CD= Cannot determine, NA= Not applicable, NR= Not reported

**Appendix 2.2: Inter-rater reliability for the Quality Assessment**

| **Criteria** | Cohen’s Kappa |
| --- | --- |
| **Quality of cross-sectional and cohort studies** |  |
| 1. Was the research question or objective in this paper clearly stated? | 1.00 |
| 2. Was the study population clearly specified and defined? | 1.00 |
| 3. Was the participation rate of eligible persons at least 50%? | 0.85 |
| 4. Were all the subjects selected or recruited from the same or similar populations (including the same time period)? Were inclusion and exclusion criteria for being in the study prespecified and applied uniformly to all participants? | 0.85 |
| 5. Was a sample size justification, power description, or variance and effect estimates provided? | 0.90 |
| 6. For the analyses in this paper, were the exposure(s) of interest measured prior to the outcome(s) being measured? | 0.80 |
| 7. Was the timeframe sufficient so that one could reasonably expect to see an association between exposure and outcome if it existed? | 1.00 |
| 8. For exposures that can vary in amount or level, did the study examine different levels of the exposure as related to the outcome (e.g., categories of exposure, or exposure measured as continuous variable)? | 0.90 |
| 9. Were the exposure measures (independent variables) clearly defined, valid, reliable, and implemented consistently across all study participants? | 1.00 |
| 10. Was the exposure(s) assessed more than once over time? | 1.00 |
| 11. Were the outcome measures (dependent variables) clearly defined, valid, reliable, and implemented consistently across all study participants? | 1.0 |
| 12. Were the outcome assessors blinded to the exposure status of participants? | 1.00 |
| 13. Was loss to follow-up after baseline 20% or less? | 1.00 |
| 14. Were key potential confounding variables measured and adjusted statistically for their impact on the relationship between exposure(s) and outcome(s)? | 0.85 |
|  |  |
| **Randomised controlled trial (RCT)** |  |
| 1. Was the study described as randomized, a randomized trial, a randomized clinical trial, or an RCT? | 1.00 |
| 2. Was the method of randomization adequate (i.e., use of randomly generated assignment)? | 1.00 |
| 3. Was the treatment allocation concealed (so that assignments could not be predicted)? | 1.00 |
| 4. Were study participants and providers blinded to treatment group assignment? | 1.00 |
| 5. Were the people assessing the outcomes blinded to the participants' group assignments? | 1.00 |
| 6. Were the groups similar at baseline on important characteristics that could affect outcomes (e.g., demographics, risk factors, co-morbid conditions)? | 1.00 |
| 7. Was the overall drop-out rate from the study at endpoint 20% or lower of the number allocated to treatment? | 1.00 |
| 8. Was the differential drop-out rate (between treatment groups) at endpoint 15 percentage points or lower? | 1.00 |
| 9. Was there high adherence to the intervention protocols for each treatment group? | 1.00 |
| 10. Were other interventions avoided or similar in the groups (e.g., similar background treatments)? | 1.00 |
| 11. Were outcomes assessed using valid and reliable measures, implemented consistently across all study participants? | 1.00 |
| 12. Did the authors report that the sample size was sufficiently large to be able to detect a difference in the main outcome between groups with at least 80% power? | 1.00 |
| 13. Were outcomes reported or subgroups analyzed prespecified (i.e., identified before analyses were conducted)? | 1.00 |
| 14. Were all randomized participants analyzed in the group to which they were originally assigned, i.e., did they use an intention-to-treat analysis? | 1.00 |
|  |  |
| **Case-Control** |  |
| 1. Was the research question or objective in this paper clearly stated and appropriate? | 1.00 |
| 2. Was the study population clearly specified and defined? | 1.00 |
| 3. Did the authors include a sample size justification? | 1.00 |
| 4. Were controls selected or recruited from the same or similar population that gave rise to the cases (including the same timeframe)? | 1.00 |
| 5. Were the definitions, inclusion and exclusion criteria, algorithms or processes used to identify or select cases and controls valid, reliable, and implemented consistently across all study participants? | 1.00 |
| 6. Were the cases clearly defined and differentiated from controls? | 1.00 |
| 7. If less than 100 percent of eligible cases and/or controls were selected for the study, were the cases and/or controls randomly selected from those eligible? | 1.00 |
| 8. Was there use of concurrent controls? | 1.00 |
| 9. Were the investigators able to confirm that the exposure/risk occurred prior to the development of the condition or event that defined a participant as a case? | 1.00 |
| 10. Were the measures of exposure/risk clearly defined, valid, reliable, and implemented consistently (including the same time period) across all study participants? | 1.00 |
| 11. Were the assessors of exposure/risk blinded to the case or control status of participants? | 1.00 |
| 12. Were key potential confounding variables measured and adjusted statistically in the analyses? If matching was used, did the investigators account for matching during study analysis? | 1.00 |

Note: Criteria from National Heart, Lung, and Blood Institute (NHLBI). (2014). Study quality assessment tools. Retrieved from https://www.nhlbi.nih.gov/health-topics/study-quality-assessment-tools

**Appendix 3: The effect size of ADHD by pooling HRQoL of children with and without ADHD**

**Appendix 3.1a: Parent-reported overall functioning**

**Appendix 3.1b: Child-reported overall functioning**

**Appendix 3.2a: Parent-reported physical functioning**

**Appendix 3.2b: Child-reported physical functioning**

**Appendix 3.3a: Parent-reported psychosocial functioning**

**Appendix 3.3b: Child-reported psychosocial functioning**

**Appendix 3.4a: Parent-reported emotional functioning**

**Appendix 3.4b: Child-reported emotional functioning**

**Appendix 3.5a: Parent-reported social functioning**

**Appendix 3.5b: Child-reported social functioning**

**Appendix 3.6a: Parent-reported school functioning**

**Appendix 3.6b: Child-reported school functioning**

**Appendix 4: The effect size of ADHD for HRQoL of children with ADHD by pooling parent and child reporting**

| Domain | Parent and child-reported | |
| --- | --- | --- |
|  | Hedges' g (95%CI) | Number of studies |
| Total | -0.49 (-0.68, -0.31) | 6 |
| Physical functioning | -0.23 (-0.37, -0.08) | 6 |
| Psychosocial functioning | -0.50 (-0.89, -0.11) | 5 |
| Emotional functioning | -0.52 (-0.79, -0.25) | 4 |
| Social functioning | -0.26 (-0.84, 0.31) | 4 |
| School functioning | -0.58 (-1.61, 0.44) | 4 |

Note: This analysis included children with ADHD.

ADHD= Attention-deficit/hyperactivity disorder, HRQoL= Health-related quality of life

**Appendix 5: The effect size of ADHD for HRQoL of children without ADHD by pooling parent and child reporting**

| Domain | Parent and child-reported |  |
| --- | --- | --- |
|  | Hedges' g (95%CI) | Number of studies |
| Total | 0.005 (-1.08, 1.09) | 6 |
| Physical functioning | -0.04 (-1.14, 1.05) | 6 |
| Psychosocial functioning | 0.04 (-1.29, 1.38) | 5 |
| Emotional functioning | 0.06 (-0.78, 0.89) | 4 |
| Social functioning | 0.03 (-1.30, 1.38) | 4 |
| School functioning | 0.01 (-0.89, 0.91) | 4 |

Note: This analysis included children without ADHD.

ADHD= Attention-deficit/hyperactivity disorder, HRQoL= Health-related quality of life

**Appendix 6: Heterogeneity test and publication bias**

| Rater | HRQoL domain | Cochran's Q | Number of studies | p-value | I^2^ | LFK index |
| --- | --- | --- | --- | --- | --- | --- |
| Parent | Total (overall) | 245.47 | 8 | P<0.05 | 97.15 | -2.17 |
|  | Physical functioning | 138.99 | 8 | P<0.05 | 94.96 | -1.20 |
|  | Psychosocial functioning | 269.02 | 8 | P<0.05 | 97.77 | -2.94 |
|  | Emotional functioning | 175.28 | 6 | P<0.05 | 97.15 | -3.91 |
|  | Social functioning | 236.59 | 6 | P<0.05 | 97.89 | -4.09 |
|  | School functioning | 233.01 | 6 | P<0.05 | 97.85 | -4.09 |
| Child | Total (overall) | 96.11 | 6 | P<0.05 | 94.80 | -1.13 |
|  | Physical functioning | 29.02 | 6 | P<0.05 | 82.77 | 1.30 |
|  | Psychosocial functioning | 97.54 | 5 | P<0.05 | 95.90 | -0.93 |
|  | Emotional functioning | 48.69 | 4 | P<0.05 | 93.84 | -2.95 |
|  | Social functioning | 96.72 | 4 | P<0.05 | 96.90 | -2.87 |
|  | School functioning | 115.77 | 4 | P<0.05 | 97.41 | -3.14 |

Note: This analysis included both children with and without ADHD.

ADHD= Attention-deficit/hyperactivity disorder, HRQoL= Health-related quality of life
